# Rare Inherited Cholestatic Disorders and Molecular Links to Hepatocarcinogenesis

**DOI:** 10.3390/cells11162570

**Published:** 2022-08-18

**Authors:** Rebecca Jeyaraj, Deirdre Kelly

**Affiliations:** 1 National Institute for Health Research Great Ormond Street Hospital Biomedical Research Centre, University College London, London WC1N 1EH, UK; 2 Institute of Immunology and Immunotherapy, University of Birmingham, Birmingham B15 2TT, UK; 3 Liver Unit, Birmingham Women’s and Children’s Hospital, Birmingham B4 6NH, UK

**Keywords:** *ABCB11* gene, *TJP2* gene, *VPS33B* gene, inherited cholestasis, hepatocellular carcinoma, hepatocarcinogenesis

## Abstract

Hepatocellular carcinoma (HCC) is the most common primary liver cancer affecting adults and the second most common primary liver cancer affecting children. Recent years have seen a significant increase in our understanding of the molecular changes associated with HCC. However, HCC is a complex disease, and its molecular pathogenesis, which likely varies by aetiology, remains to be fully elucidated. Interestingly, some inherited cholestatic disorders that manifest in childhood are associated with early HCC development. This review will thus explore how three genes that are associated with liver disease in childhood (*ABCB11, TJP2* and *VPS33B*) might play a role in the initiation and progression of HCC. Specifically, chronic bile-induced damage (caused by *ABCB11* changes), disruption of intercellular junction formation (caused by *TJP2* changes) and loss of normal apical–basal cell polarity (caused by *VPS33B* changes) will be discussed as possible mechanisms for HCC development.

## 1. Introduction

Inherited cholestatic disorders refer to genetically determined conditions in which there is a defect in bile synthesis, secretion or flow. These conditions generally result in increased serum concentrations of the various components of bile, may manifest clinically as jaundice, pruritus, as well as fat and fat-soluble vitamin malabsorption, and can lead to significant liver injury. Certain inherited cholestatic disorders are associated with an increased risk of liver cancer in infancy and childhood. The exact mechanism by which cancer develops in patients with these disorders is unclear. However, their monogenic nature and the early onset of cancer in these disorders, typically in the absence of other known environmental factors, could shed light on how specific genes contribute to hepatocarcinogenesis. This review will thus consider how new insights into genes associated with rare paediatric cholestatic disorders might improve our understanding of the most common liver cancer type affecting adults—that is, hepatocellular carcinoma (HCC).

The review will first provide a brief overview of the types of primary liver cancer affecting children and adults. Next, it will summarise the key similarities and differences between paediatric and adult HCC. Finally, three genes implicated in rare cholestatic disorders will be discussed in relation to the development of HCC, focusing in particular on bile acid-induced damage (due to ATP-binding cassette subfamily B member 11, or *ABCB11*, changes), disruption of intercellular junction formation (due to tight junction protein 2, or *TJP2*, changes) and disruption of cell polarity (due to vacuolar protein sorting-associated protein 33B, or *VPS33B*, changes). The review focuses specifically on the *ABCB11, TJP2* and *VPS33B* genes due to the important and increasingly well-defined mechanisms by which variants in these genes lead to cholestasis and possibly HCC.

## 2. Primary Liver Cancer in Children and Adults

In adults, HCC accounts for approximately 75–90% of all liver cancers [1,2]. Major risk factors for HCC in adults include chronic viral hepatitis, alcohol-related liver disease and metabolic syndrome-associated liver disease [3,4]. The majority of HCCs occur in cirrhotic livers, although they may also occur in noncirrhotic livers. After HCC, the second most common type of liver cancer in adults is cholangiocarcinoma (CCA). Important risk factors for CCA include parasitic infection, primary sclerosing cholangitis, bile duct cysts and cholelithiasis [5]. A small subset of primary liver cancers exhibit features of both HCC and CCA and are referred to as combined hepatocellular-cholangiocarcinoma [6].

In children, hepatoblastoma is the most common type of liver cancer, although it accounts for just 1% of all paediatric cancers. Hepatoblastoma is associated with certain genetic syndromes such as Beckwith–Wiedemann syndrome, familial adenomatous polyposis and trisomy 18 [7,8]. Prematurity and low or very low birth weight have also been associated with hepatoblastoma [7,8] (Table 1). HCC is the second most common type of liver cancer in children and is the most common liver cancer diagnosed in children with inherited metabolic disorders [9]. Risk factors for HCC in children include genetically determined disorders such as progressive familial intrahepatic cholestasis (PFIC), hereditary tyrosinemia, glycogen storage disease (GSD), Alagille’s syndrome, as well as other conditions such as perinatally acquired hepatitis B virus (HBV) infection and congenital portosystemic shunts (Table 1). Some rare tumours show overlapping histological features between HCC and hepatoblastoma; these tumours, which typically affect older children and adolescents, were once termed ‘transitional liver cell tumours’ but are now by consensus termed ‘hepatocellular malignant neoplasms, not otherwise specified’ [10,11,12]. Fibrolamellar carcinoma (FLC) is another rare type of liver cancer that primarily affects adolescents and young adults without pre-existing chronic liver disease. Risk factors for its development have yet to be firmly established [13]. Though traditionally considered a variant of HCC, it has recently been suggested that FLC is likely to represent a distinct clinicopathologic entity [14]. CCA is also rare in children but has been described in the context of underlying liver [15,16] or immunological disease [17].

Other hepatic neoplasms include angiosarcoma of the liver and benign lesions such as hepatic adenoma and focal nodular hyperplasia. This review will focus on HCC from here on, given the comparatively larger number of people affected by this condition in the general population.

## 3. Differences between Paediatric and Adult HCC

The cell of origin in paediatric and adult HCC has been subject to significant debate and study. It is possibly the hepatocyte, a specialised epithelial cell in the liver that performs myriad metabolic, synthetic, detoxification and secretory functions; however, the hepatic progenitor cell has also previously been suggested as a potential cell of origin [40,41]. Given the different risk factors associated with HCC development in children and in adults, it is worth considering at this stage whether paediatric HCC and adult HCC are, in fact, the same disease.

There are some general differences between paediatric and adult cancers. For instance, paediatric cancers generally tend to exhibit fewer genetic alterations compared to adult cancers [42]. Some of the proposed reasons for the lower mutational burden seen in paediatric cancers include their embryonal origin in many cases, perturbance of normal developmental pathways, and in the liver, the higher basal cellular growth rate in the paediatric liver [42,43]. Environmental carcinogens also seem to play a smaller role in paediatric cancers in general [42,44], although it must be acknowledged that there are certain well-established associations between environmental factors and childhood cancer—for instance, perinatally acquired HBV infection and the early development of HCC.

With respect to HCC, it is notable that response rates to chemotherapy are estimated to be approximately 20% in adults and 40% in children [45]. Different chemotherapeutic regimes and the higher frequency of medical comorbidities in adults should be taken into account when considering these different response rates in children and adults. At the same time, the significant difference in chemo-responsiveness may also be biologically determined and may suggest disparate mechanisms of HCC development.

Another difference between paediatric and adult HCC relates to the presence of underlying liver disease. While there are known risk factors for HCC in children, the majority of HCC cases are sporadic, occurring in the absence of pre-existing liver disease. In contrast, while HCC in adults can develop independently of cirrhosis, the majority of HCC cases occur in the setting of cirrhosis. Moreover, in the paediatric population, there is no clearly established difference in HCC risk between males and females [46]. This contrasts with the adult population, in which HCC occurs more frequently in men than in women [47].

Although large-scale studies to clarify the molecular and histopathological differences between paediatric and adult HCCs are inevitably limited by the rarity of paediatric HCC, there are some notable differences. For example, one group studying HBV-related HCC found a lower level of cyclin D1 expression and a higher frequency of loss of heterozygosity of chromosome 13q in paediatric tumours compared to adult tumours [48]. The significance of loss of heterozygosity of chromosome 13q may relate to loss of function of a tumour suppressor gene in this region, such as the retinoblastoma (*RB*) gene, the BRCA2 DNA repair associated (*BRCA2*) gene or another putative gene [49,50]. Another group reported diffuse expression of epithelial cell adhesion molecule (EpCAM) in 11 out of 12 (92%) paediatric HCCs compared to only focal EpCAM expression in 3 out of 20 (15%) adult HCCs [51]. It was suggested that the particular expression pattern of EpCAM in paediatric HCCs might reflect cell immaturity [51]. Although the exact implications of diffuse versus focal EpCAM expression are not certain, EpCAM has been described in the context of multiple cancers as a marker of cell ‘stemness’ [52,53].

While these differences are interesting (summarised in Table 2), there are also important parallels between paediatric and adult HCC. For instance, conventional paediatric HCC and adult HCC currently share diagnostic histological findings such as increased cell density, cytologic and nuclear atypia, frequent mitotic activity, loss of reticulin, increased arterialisation and expansion of the hepatocyte plate [54,55]. In addition, cellular pathways involved in growth, differentiation, apoptosis, angiogenesis and cell cycle control are frequently dysregulated in both paediatric and adult patients with HCC [43]. The genetic alterations present in paediatric and adult cancers are also highly heterogeneous [42]. For example, adult cancers show a high frequency of somatic mutations, and paediatric cancers may exhibit a higher frequency of germline changes, copy number alterations, structural chromosomal rearrangements such as chromoplexy, gene fusions and enhancer hijacking [42]. In a more clinical context, elevation of serum alpha-fetoprotein (AFP) levels may be seen in many but not all cases of paediatric and adult HCC [18,56,57,58], and many institutions use ultrasound imaging with monitoring of serum AFP levels as a screening or surveillance method for at-risk patients in both paediatric and adult populations. Finally, several genes that are associated with severe paediatric liver disease and/or early-onset HCC have also been implicated in adult HCC. The next section will thus focus on how insights into three genes associated with rare cholestatic disorders (*ABCB11, TJP2* and *VPS33B*) might clarify the molecular events leading to the development and progression of adult-type HCC.

## 4. *ABCB11* and Bile Acid-Induced Damage

*ABCB11* encodes the bile salt export pump (BSEP), which facilitates the rate-limiting step in the transport of bile acids from portal blood into bile by transporting conjugated bile acids across the hepatocyte canalicular membrane. Biallelic mutations in *ABCB11* can cause PFIC type 2 (PFIC 2), which is characterised by BSEP deficiency, intracellular retention of bile salts and severe cholestatic disease [59]. Patients with PFIC 2 have a 5–10% risk of developing HCC by 2 years of age. The association between *ABCB11* mutations and HCC in these patients may relate to chronic cholestasis and inflammation, which provides a milieu for the development of cancer. Although not a focus of this review, early-onset CCA has also been observed in patients with PFIC 2 [60,61].

In general terms, chronic cholestasis can lead to bile acid accumulation in the hepatocyte and subsequent liver injury through several putative mechanisms. For example, bile acid-induced liver injury was traditionally thought to result from a detergent action on cells, but more recently, the induction of inflammatory cytokines as well as the recruitment and cytotoxic action of neutrophils and other innate immune cells are thought to be of greater relevance [62]. A chronic necroinflammatory state then leads to fibrosis and cirrhosis, which provides the setting for dysplastic nodules and HCC to develop [63]. However, this sequence of events does not explain why children with other cholestatic disorders do not show the same predisposition to HCC development, nor why the time between the onset of liver disease and HCC development is particularly short in some BSEP-deficient patients. It is possible that the risk of HCC is related to the severity of BSEP deficiency and, thereby, the severity of inflammatory damage; for instance, in a retrospective cohort study, only 3% of patients with less severe *ABCB11* mutations developed HCC by 15 years of age compared with 34% of patients with more severe *ABCB11* mutations (i.e., mutations leading to nonfunctional or absent BSEP protein) [64].

Iannelli et al. found that the genomic landscape of human HCCs induced by defects in BSEP (referred to as BSEP-HCCs) appeared to be distinct from that of HCCs related to viral infection or alcohol [65]. In their study, human BSEP-HCC genomes showed relatively few point mutations in known cancer-causing genes compared with other human HCCs and paediatric cancers. However, BSEP-HCCs did show massive gene amplifications and rearrangements affecting the mitogen-activated protein kinase (MAPK), ErbB and PI3K/Akt signalling pathways. The authors suggested that these amplifications likely occurred through stepwise rearrangements rather than through single catastrophic events because signs of chromothripsis were not frequent. They further suggested that c-Jun N-terminal kinase (JNK) amplification, in particular, seemed to favour tumour progression in BSEP-HCCs [65].

This study on HCC in the context of BSEP deficiency offers several learning points about HCC in general. First, the authors proposed that external mutagens are the likely cause of mutational instability in HCC, given the comparatively few point mutations they found in BSEP-HCCs [65]. Second, the study emphasises that HCCs are a heterogeneous group with genomic changes differing based on aetiology. This is of particular importance, as future targeted therapies for HCC may be developed on the basis of the specific genomic changes seen with each aetiology. In this same study, for instance, mice deficient for *Mdr2* (who are thought to develop chronic liver disease through a sequence of events similar to that seen in human BSEP deficiency) were treated with a pharmacological inhibitor of JNK [65]. After 3 weeks of pharmacological inhibition, liver nodules from treated *Mdr2*-deficient mice appeared to be smaller, with lower HCC content in each nodule compared with untreated *Mdr2*-deficient mice, although there were a similar number of nodules in each group. The authors consequently posited that JNK inhibition may delay the progression of HCC in BSEP-deficient patients and that the JNK pathway may represent a possible future target for therapeutic intervention [65]. It is worth noting, however, that the role of JNK in the liver is complex. JNK is also known to play a role in liver regeneration following partial hepatectomy in animal models, for instance [66,67,68]. Further work is thus needed to clarify whether JNK inhibition is, in fact, a safe therapeutic strategy or whether unintended effects are likely to undermine its clinical benefit.

Interestingly, a case report of HCC in a 17-month-old patient with biallelic germline *ABCB11* mutations found somatic mutations in the cancer-related genes beta-catenin (*CTNNB1*) and nuclear factor erythroid 2-related factor 2 (*NFE2L2*). Moreover, the background liver parenchyma in this patient did not appear to show inflammatory or fibrotic changes, although abnormal cytokeratin-7 expression on histological evaluation suggested chronic cholestasis [69]. The authors suggested that the *ABCB11* loss-of-function mutations may have set the stage for *CTNNB1* and *NFE2L2* somatic mutations to arise in this case. This report is thus a useful reminder that even within the select group of patients with BSEP deficiency, HCC can be a heterogenous entity occurring both in the presence or absence of background cirrhosis and with variable somatic genetic changes.

The recent licensing of certain ileal bile acid transporter (IBAT) inhibitors may lead to important changes in the long-term outlook of patients with PFIC 2, particularly in relation to the risk of HCC development. Odevixibat, for instance, is a small molecule oral IBAT inhibitor that works by reducing the enterohepatic circulation of bile acids. As almost 95% of bile acids are reabsorbed in the ileum via the enterohepatic circulation, IBAT inhibitors such as odevixibat, which block this step, should lead to a reduction in serum bile acid levels [70]. In the long term, a sustained reduction in serum bile acid levels in these patients could limit bile acid toxicity to the liver and perhaps reduce the risk of HCC if HCC in these cases is, in fact, driven by chronic bile acid-induced toxicity. Importantly, however, odevixibat may not necessarily be effective in patients with severe truncating *ABCB11* mutations who have a severe deficiency or absence of the BSEP protein and who are also at higher risk of HCC [64,71]. Moreover, it may be that pharmacological therapy needs to be instituted early in the disease course before premalignant or malignant cells develop in order to have a real impact on long-term HCC risk in BSEP-deficient patients. Therefore, although diagnostic delays are generally common in rare genetic disorders, the availability of new effective therapies makes it increasingly important that patients with PFIC who are eligible for IBAT inhibitors are promptly identified to maximise any possible reduction in HCC risk. Similarly, where the IBAT inhibitor maralixibat is used in patients with Alagille’s syndrome, the time point at which this pharmacological option is commenced may be important for controlling the HCC risk, particularly since the HCC risk in Alagille’s syndrome is thought to be related to the development of cirrhosis. Ultimately, long-term studies are needed to clarify the impact of these newly licensed IBAT inhibitors on HCC risk in BSEP-deficient patients as well as other patient groups at risk of HCC.

## 5. *TJP2* and Disruption of Intercellular Junction Formation

*TJP2* encodes the zona occludens 2 (ZO-2) protein, which stabilises tight junctions between cells by linking the cytoplasmic termini of transmembrane proteins to the actin cytoskeleton. Biallelic mutations in *TJP2* cause progressive familial intrahepatic cholestasis type 4 (PFIC 4), and an association between PFIC 4 and early HCC development has been reported [31,72]. The disruption of intercellular junctions in PFIC 4 leads to the paracellular leak of biliary contents into the liver parenchyma and chronic bile-induced injury, which may be the underlying mechanism for carcinogenesis in PFIC 4. However, other chronic cholestatic conditions in which there is chronic bile-induced injury do not appear to confer the same risk of HCC as is seen in PFIC 4.

It has been suggested that *TJP2* may, in fact, have a role as a tumour suppressor gene. One group found that *TJP2* expression was downregulated in chronic liver disease, HCC and CCA compared with pathological control tissue [73]. Moreover, in their study, most ZO-2 protein was found at the cellular junctions of biliary epithelial cells and hepatocytes in pathological control and chronically diseased hepatic tissue, while most ZO-2 protein was found in the perinuclear region in HCC and CCA tissue. These findings suggest important differences in *TJP2* expression and protein localisation in HCC compared with noncancerous liver tissue [73]. Aberrant expression of *TJP2* has also been noted in nonliver cancers. For instance, aberrant methylation of *TJP2* was observed in 70% of primary pancreatic cancers in one study [74]. However, correlation does not imply causation, and the mere association between aberrant *TJP2* expression and cancer is not in itself evidence of a tumour suppressor effect.

Instead, González-Mariscal et al. highlighted (i) the structural similarity between ZO-2 and a known tumour suppressor protein in *Drosophilia*, (ii) the targeting of ZO-2 by viral oncogenic proteins in animal models and (iii) the role of ZO-2 as a transcriptional repressor of certain genes involved in cell proliferation as possible evidence for a tumour suppressor effect [75]. Moreover, tight junctions are now known to act as a crucial site for the regulation of cell proliferation and differentiation by facilitating interactions between junctional proteins, the actin cytoskeleton and other signalling proteins [76,77]. Other authors have gone on to support the notion that ZO-2 may have a junction-unrelated role, in which nuclear localisation of ZO-2 allows it to act as a nuclear factor affecting gene expression and cell growth and proliferation [78,79]. For example, ZO-2 has been shown to interact with transcriptional enhanced associate domain (TEAD) transcription factors [79], with deregulation of the latter affecting important signal transduction pathways implicated in tumour progression and cancer metabolism [80].

However, not all people with PFIC 4 develop HCC. For instance, Wei et al. described a family in which five siblings who were homozygous for the same *TJP2* mutation showed variable degrees of liver disease; for instance, one sibling developed HCC at the age of 23, one sibling developed acute-on-chronic liver failure at the age of 36 without evidence of HCC, one sibling developed severe intrahepatic cholestasis of pregnancy and two other siblings showed elevated liver enzymes at the ages of 19 and 25 without evidence of chronic liver disease at the time [81]. This suggests that other factors are likely to work in combination with the *TJP2* mutation to contribute to HCC development in these patients.

When considering the possible impact of IBAT inhibitors in PFIC 4 patients, it is important to note that the majority of data on IBAT inhibitors initially focused on PFIC type 1 and PFIC 2. However, the open-label PEDFIC2 study included patients with any PFIC type, and odevixibat has now been approved for use in all PFIC types [71,82]. It will be interesting to see whether future long-term studies are able to uncover any effect of IBAT inhibitors on HCC risk in patients with PFIC 4. If a reduction in HCC risk is found, this may suggest that at least part of the HCC risk in these patients could also relate to bile acid toxicity, perhaps in a similar way to that described in Section 4 for PFIC 2 patients with *ABCB11* changes.

## 6. *VPS33B* and Disruption of Cell Polarity

*VPS33B* encodes the vacuolar protein sorting-associated protein 33B, which plays a role in vesicle trafficking and the maintenance of apical–basal cell polarity. Biallelic mutations in *VPS33B* cause arthrogryposis, renal dysfunction and cholestasis (ARC) syndrome [83]. There are no published reports confirming an association between ARC syndrome and early HCC development in affected patients, although such an association may be obscured by the rarity of ARC syndrome or the shortened life expectancy of patients with the condition. However, preclinical studies consistently suggest an important role for *VPS33B* in carcinogenesis.

Loss of *VPS33B* has been associated with hepatocarcinogenesis in a *VPS33B*-knockout mouse model, attributable to loss of a tumour suppressor effect [84]. In this study, *VPS33B* deficiency appeared to lead to a decrease in E-cadherin expression at the lateral plasma membrane despite preserved mRNA levels. Instead, E-cadherin was found to be located to lysosomes in *VPS33B*-deficient mouse livers, suggesting that *VPS33B* may have a role in the post-translational regulation and accurate subcellular localisation of E-cadherin. Loss of E-cadherin, particularly in combination with Ras activation or a chemical carcinogen, has been previously associated with accelerated hepatocarcinogenesis and an invasive cancer phenotype characterised by epithelial–mesenchymal transition and upregulated stem cell markers [85].

Lower *VPS33B* expression in the HCC tissue of patients has also been associated with poor pathologic differentiation and more satellite nodes compared with those with higher or preserved *VPS33B* expression [84]. Furthermore, lower *VPS33B* expression has been associated with shorter disease-free survival and overall survival in patients with HCC [84]. Taken together, these findings suggest that *VPS33B* may have a role not only in the initial development of HCC but also in its progression and in overall patient prognosis.

The likely role of *VPS33B* in cancer progression and invasiveness is not limited to the liver. In patients with renal cell carcinoma, higher levels of *VPS33B* expression were found to be independently and positively associated with recurrence-free survival [86]. Overexpression of *VPS33B* has been reported to decrease cell proliferation and chemoresistance in in vitro assays and in vivo mouse studies of lung adenocarcinoma [87], nasopharyngeal carcinoma [88], colorectal cancer [89] and ovarian cancer [90]. This inhibitory effect on proliferation was attributed to VPS33B-mediated modulation of the epidermal growth factor receptor (EGFR) signalling and other pathways. Cell migration and invasion, as well as intrahepatic dissemination and lung metastasis, were also lower when *VPS33B* was experimentally overexpressed in cell and mouse models of lung adenocarcinoma [87].

Tissue architecture and the intracellular signalling pathways that govern cell proliferation, invasion and metastasis are increasingly thought to be dependent on the reliable specification of apical and basolateral membrane domains within the cell [91]. Disrupted cell polarity is thus now widely recognised as a cause, rather than only a consequence, of dysregulated growth and proliferation. Genes with a role in cell polarisation, such as *VPS33B*, could be key players in carcinogenesis, and further molecular studies would be useful to clarify how cellular spatial asymmetry and morphology become disrupted and promote the initiation and progression of cancer in cells, tissues and/or patients with a loss of VPS33B.

## 7. Conclusions

The multihit model of cancer development suggests that multiple changes need to occur before a cell becomes cancerous. The finding that certain genes that cause rare paediatric cholestatic disorders are also associated with HCC development and/or progression can shed light on some of the key mechanistic steps that lead to a cell acquiring a cancerous phenotype. For example, *ABCB11* mutations may cause chronic bile-induced inflammation and increase the risk of dysplasia and neoplasia. *TJP2* mutations may increase the risk of HCC through junction-related effects (as tight junctions can regulate cell growth and proliferation) or through junction-unrelated effects (as the encoded ZO-2 protein may serve as a regulator of gene expression). Changes in *VPS33B* expression may interfere with intracellular vesicle trafficking and, therefore, the establishment of apical–basal cell polarity, leading to deregulated cell signalling and proliferation. However, further work is needed to understand why not all patients with loss-of-function mutations in these genes develop HCC, how the presence or absence of background liver disease influences the risk of HCC and how mutations in the abovementioned genes work in concert with other mutations to drive cancer progression. It is also worth considering that perhaps no single molecular change, in itself, is necessary or sufficient to cause HCC, given the heterogeneity of the condition. Future studies to investigate the effect of newly licensed IBAT inhibitors on long-term HCC risk in patients with genetic cholestasis will provide additional insights into how HCC development might be affected by serum bile acid levels. In the meantime, it is essential to remain vigilant in detecting HCC in genetically primed patients through regular ultrasound imaging, serum AFP monitoring and magnetic resonance imaging where indicated. Ultimately, a better understanding of HCC development across different patient groups could help identify new druggable molecular targets, focus preventive and therapeutic efforts, and improve patient outcomes.

## Figures and Tables

**Table 1:** Risk factors for hepatoblastoma and HCC in children [7,8,9,18,19,20,21,22,23,24,25,26,27,28,29,30,31,32,33,34,35,36,37,38,39].

Type of Primary Liver Cancer	Risk Factors (Not Exhaustive)	References
**Hepatoblastoma**	**Genetic, overgrowth and/or cancer predisposition syndromes** Beckwith–Wiedemann syndrome Familial adenomatous polyposis Trisomy 18 Aicardi syndrome Simpson–Golabi–Behmel syndrome **Other conditions** Prematurity Low or very low birth weight	[7,8]
**HCC**	**Possibly related to cirrhosis** Alagille’s syndrome Hereditary tyrosinemia GSD type 3 Alpha-1 antitrypsin deficiency Transaldolase deficiency Liver mitochondrial respiratory chain disorders Wilson’s disease Autoimmune liver disease Biliary atresia **Possibly independent of cirrhosis** PFIC GSD type 1 Citrin deficiency (including adult-onset citrullinemia type 2) Congenital portosystemic shunts Perinatally acquired HBV infection and hepatitis C virus (HCV) infection Hepatic venous outflow tract obstruction Note: Some conditions that can affect children (e.g., acute intermittent porphyria, hereditary haemochromatosis, chronic HCV infection, nonalcoholic fatty liver disease) may increase the risk of HCC later in life due to a longer latency period.	[9,18,19,20,21,22,23,24,25,26,27,28,29,30,31,32,33,34,35,36,37,38,39]

Abbreviations: GSD, glycogen storage disease; PFIC, progressive familial intrahepatic cholestasis; HBV, hepatitis B virus; HCV, hepatitis C virus; HCC, hepatocellular carcinoma.

**Table 2:** Summary of differences between paediatric and adult HCCs [3,4,18,33,42,43,44,45,48,51].

Paediatric Cancers	Adult Cancers	Reference
**In General**		
Fewer genetic alterations	More frequent genetic alterations	[42]
Environmental factors play a smaller role	Environmental factors play a greater role	[42,44]
No clear difference in risk between males and females	Risk is higher in males compared to females	[46,47]
**Focus on HCC**		
Majority of HCC cases are sporadic	Majority of HCC cases occur in the setting of cirrhosis	[33]
Risk factors include genetic cholestatic conditions (e.g., PFIC, Alagille’s syndrome), metabolic liver disease (e.g., hereditary tyrosinemia, GSD), perinatal HBV infection and congenital portosystemic shunts	Major risk factors include chronic viral hepatitis, alcohol-related liver disease and metabolic syndrome-associated liver disease	[3,4,18,33]
Higher response rates to chemotherapy	Lower response rates to chemotherapy	[45]
Lower level of cyclin D1 expression and higher frequency of loss of heterozygosity of chromosome 13q in HBV-related HCCs	Higher level of cyclin D1 expression and lower frequency of loss of heterozygosity of chromosome 13q in HBV-related HCCs	[48]
Diffuse EpCAM expression in the majority of cases in one study	Focal EpCAM expression in some cases in one study	[51]

Abbreviations: HCC, hepatocellular carcinoma; PFIC, progressive familial intrahepatic cholestasis; GSD, glycogen storage disease; HBV, hepatitis B virus; EpCAM, epithelial cell adhesion molecule.

## Data Availability

Not applicable.

## Abbreviations

***ABCB11***	ATP-binding cassette subfamily B member 11
**AFP**	Alpha-fetoprotein
**ARC syndrome**	Arthrogryposis, renal dysfunction and cholestasis syndrome
***BRCA2***	BRCA2 DNA repair associated
**BSEP**	Bile salt export pump
**CCA**	Cholangiocarcinoma
***CTNNB1***	Beta-catenin
**EpCAM**	Epithelial cell adhesion molecule
**FLC**	Fibrolamellar carcinoma
**GSD**	Glycogen storage disease
**HBV**	Hepatitis B virus
**HCC**	Hepatocellular carcinoma
**HCV**	Hepatitis C virus
***IBAT***	Ileal bile acid transporter
**JNK**	c-Jun N-terminal kinase
**MAPK**	Mitogen-activated protein kinase
***NFE2L2***	Nuclear factor erythroid 2-related factor 2
**PFIC**	Progressive familial intrahepatic cholestasis
**PFIC 2**	Progressive familial intrahepatic cholestasis type 2
**PFIC 4**	Progressive familial intrahepatic cholestasis type 4
***RB***	Retinoblastoma
***TEAD***	Transcriptional enhanced associate domain
***TJP2***	Tight junction protein 2
***VPS33B***	Vacuolar protein sorting-associated protein 33B
